# Rotavirus Replication in the Cholangiocyte Mediates the Temporal Dependence of Murine Biliary Atresia

**DOI:** 10.1371/journal.pone.0069069

**Published:** 2013-07-03

**Authors:** Sujit K. Mohanty, Bryan Donnelly, Alexander Bondoc, Mubeen Jafri, Ashley Walther, Abigail Coots, Monica McNeal, David Witte, Gregory M. Tiao

**Affiliations:** 1 Department of Pediatric and Thoracic Surgery, Cincinnati Children’s Hospital Medical Center, Cincinnati, Ohio, United States of America; 2 Division of Infectious Diseases, Cincinnati Children’s Hospital Medical Center, Cincinnati, Ohio, United States of America; 3 Department of Pathology, Cincinnati Children’s Hospital Medical Center, Cincinnati, Ohio, United States of America; Institute of Hepatology, Foundation for Liver Research, United Kingdom

## Abstract

Biliary atresia (BA) is a neonatal disease that results in obliteration of the biliary tree. The murine model of BA, which mirrors the human disease, is based upon infection of newborn mice with rhesus rotavirus (RRV), leading to an obstructive cholangiopathy. The purpose of this study was to characterize the temporal relationship between viral infection and the induction of this model. BALB/c mice were infected with RRV on day of life (DOL) 0, 3, 5, and 7. Groups were characterized as early-infection (infection by DOL 3) or late-infection (infection after DOL 5). Early RRV infection induced symptoms in 95% of pups with a mortality rate of 80%. In contrast, late infection caused symptoms in only 50% of mice, and 100% of pups survived. The clinical findings correlated with histological analysis of extrahepatic biliary trees, cytokine expression, and viral titers. Primary murine cholangiocytes isolated, cultured, and infected with RRV yielded higher titers of infectious virus in those harvested from DOL 2 versus DOL 9 mice. Less interferon alpha and beta was produced in DOL 2 versus DOL 9 RRV infected primary cholangiocytes. Injection of BALB/c interferon alpha/beta receptor knockout (IFN-αβR^−/−^) pups at DOL 7 showed increased symptoms (79%) and mortality (46%) when compared to late infected wild type mice. In conclusion, the degree of injury sustained by relatively immature cholangiocytes due to more robust RRV replication correlated with more severe clinical manifestations of cholangiopathy and higher mortality. Interferon alpha production by cholangiocytes appears to play a regulatory role. These findings confirm a temporal dependence of RRV infection in murine BA and begin to define a pathophysiologic role of the maturing cholangiocyte.

## Introduction

Biliary atresia (BA) is a devastating inflammatory cholangiopathy, leading to obstruction of the biliary tree and, without treatment, death within two years of birth. It is the most common etiology of obstructive neonatal cholestasis and remains the number one indication for pediatric liver transplantation in the United States. The incidence of BA is estimated at 1 in 8,000-15,000 live births [1] and is a disease unique to infancy. Afflicted children manifest symptoms of biliary obstruction including jaundice, acholic stool, and failure to thrive in the first few weeks of life. Initial therapy includes surgical bypass; however, most patients ultimately require liver transplantation due to end-stage disease. Interestingly, BA does not recur after transplantation.

Biliary atresia’s etiology remains uncertain. The most convincing evidence favors infectious and/or immune etiologies. Multiple viruses have been found within the livers of afflicted children, including reovirus type 3 and group C rotavirus [2–4], and studies suggest that the pathogenesis of bile duct obstruction is mediated by a T-cell, NK cell, and macrophage dependent response [5–7]. Additionally, both human and murine studies of BA have demonstrated elevated levels of inflammatory cytokines including interferon-γ (IFN-γ), tumor necrosis factor α (TNF-α), interleukin-6 (IL-6), IL-10, and IL-18 that correspond to the previously described immune response [8,9]. A unifying hypothesis has been proposed, postulating that a primary infectious agent tropic for the biliary epithelial cell, or cholangiocyte, initiates a secondary, immune-mediated injury resulting in a sclerosing cholangiopathy [10]. The murine model of BA in which infection of newborn mice with rhesus rotavirus (RRV) results in biliary obstruction is important evidence supporting this hypothesis. The morphologic and histological findings in the diseased murine extra-hepatic biliary tree are similar to those found in humans at the time of diagnosis [11,12]. In earlier work, it has been shown that RRV specifically targets the biliary epithelium for infection [13,14] and RRV’s ability to bind to, and subsequently infect, the cholangiocyte cell surface is mediated in part by the α2β1 integrin [15–19].

Previously, both Riepenhoff-Talty and Czech-Schmidt have demonstrated that the murine model of BA is induced when viral infection occurs within 48 hours of birth [20,21]. If infected after this time period, symptoms of biliary obstruction are mitigated. Other work has demonstrated that RRV infection elicits a specific, time dependent activation of apoptosis in cholangiocytes [22] and induction of regulatory T cells [23]. When taken together, these data may explain why BA occurs only in infancy, supporting the hypothesis of a perinatal trigger. The purpose of the present study was to further characterize the temporal nature of BA, focusing specifically on the interaction of the neonatal cholangiocyte with RRV as well as the subsequent severity of biliary injury.

## Materials and Methods

### Ethics Statement

All animal research was performed in accordance with regulations and protocols approved by the Institutional Animal Care and Use Committee (IACUC) at Cincinnati Children’s Hospital Medical Center.

### Murine model of biliary atresia

Breeding pairs of wild-type BALB/c mice (Harlan Labs, Indianapolis, IN) and interferon alpha/beta receptor knockout (IFN-αβR^−/−^) mice on a BALB/c background (a generous gift from Dr. Bezerra’s lab, Cincinnati Children’s Hospital Medical Center) were kept in micro-isolator cages in a virus-free environment with free access to sterilized chow and water. The mice were bred and separated approximately 1 week before their expected delivery. Intraperitoneal (i.p.) injection of newborn pups with RRV at a dose of 1.25 x 10^6^ FFU/gram body weight was performed on day of life (DOL) 0, 3, 5, or 7. Pups injected with normal saline served as controls at each time point.

Injected pups were monitored for 21 days. Weight gain, clinical signs of hepatobiliary injury (i.e. jaundice in non-fur covered skin, acholic stools and bilirubinuria), and survival were recorded. Pups categorized as symptomatic had at least two signs of cholestasis including jaundice, bilirubinuria, weight loss, acholic stools, or oily appearance of fur. Classification as “symptom-free” signified absence of any symptoms.

Subsets of infected pups were sacrificed 1, 3, 5, 7, and 14 days post-infection (PI). The gross morphology of the liver and the hepatobiliary system was documented. The extrahepatic biliary trees and a portion of the liver were procured using micro dissection. Both specimens were weighed, homogenized in 0.5 ml of Earle’s balanced salt solution (EBSS) (Invitrogen, Carlsbad, CA) supplemented with Ca^2+^ and placed on ice. Samples were stored at -80^°^C until analyzed for live virus. Additional murine bile duct and liver samples were snap frozen in Histo Prep (Fisher Scientific, Fair Lawn, New Jersey) for immunohistochemistry or were embedded in paraffin for histological analysis.

### Isolation of primary murine cholangiocytes

On DOL 2 and 9, primary cholangiocytes were harvested from non-infected neonatal BALB/c pups. DOL 2 and 9 were selected for harvest, as mice two days post-infection (injected on either DOL 0 or 7) were found to contain significant amounts of RRV in their extrahepatic biliary system, indicating that in vivo, virus had reached the biliary system and could infect cholangiocytes. Livers were harvested, homogenized, and digested in Dulbecco’s Modification of Eagle’s Medium (DMEM/F12) supplemented with L-glutamine, penicillin streptomycin, heat inactivated fetal bovine serum (FBS) (all from Invitrogen), collagenase, hyaluronidase and DNase I (all from Sigma-Aldrich, St. Louis, MO). After incubation for 1 hour at 37°C, the slurry was passed through a 40-µm filter and cells were washed, collected and purified through a Percoll gradient (GE Healthcare, Sweden). Cells were washed twice and pelleted. The cells were then resuspended in phosphate buffered saline (PBS) (Gibco) supplemented with 0.1% bovine serum albumin (BSA) and incubated at 4°C with an Ep-CAM antibody generated by the Developmental Studies Hybridoma Bank at the University of Iowa. The cell were washed, suspended in PBS/0.1% BSA and incubated at 4°C with sheep anti-rat Dynabead IgG antibodies (Invitrogen). The dynabead coated cells were further purified using a magnet, suspended in cholangiocyte growth media, plated on collagen coated T25 cell culture flasks (Becton Dickinson, Franklin Lakes, NJ) and incubated at 37°C in 5% CO_2_ until 75% confluent. The cells were passaged once and grown to confluence. During passaging, cells were regularly assayed for the cholangiocyte markers CK-7 and CK-19, and those used for experimentation came from passage 3 through 5 as described previously [24].

### Morphologic assessment of biliary injury and immunohistochemistry for viral localization

Paraffin embedded liver and bile duct samples were serially sectioned at 5µm along the length of the block. Sections were stained with hematoxylin and eosin (H&E) and Mason’s trichrome using standard techniques. For the extra-hepatic biliary tract, a previously described ordinal scoring system was used to assess the degree of injury [13]. All sections were graded by the same blinded pathologist (David Witte).

Similarly, frozen samples of liver and bile ducts were sectioned serially at 5µm. They were blocked with 2% normal goat serum and mouse-on-mouse (MOM) blocking reagent (Vector Laboratories, Burlingame, CA) for 60 min at room temperature and underwent dual immunofluorescence staining for localization of RRV to the cholangiocyte using techniques previously described [25]. All sections were analyzed using Nikon Eclipse E600 microscope and photographed with a Nikon Digital Camera DXM1200F.

### Quantification of Liver Enzymes

BALB/c pups were injected with RRV or saline on either DOL 0 or 7. Cardiac puncture was performed on subsets of mice at 7 and 12 days post injection. Serum was collected into serum separator tubes (Becton Dickinson) and 4-6 mice were pooled per sample. Quantitative determination of direct bilirubin, alanine aminotransferase (ALT) and aspartate aminotransferase (AST) were performed in triplicate as per the manufacturer instructions (Teco Diagnsotics, Anaheim, CA).

### RNA isolation and real time PCR for cytokines

BALB/c pups were injected with RRV or saline on either DOL 0 or 7. Subsets of mice were sacrificed at 2, 5, and 7 days after injection, their livers were harvested, and the messenger RNA (mRNA) expression for IFN-γ, TNF-α, IL-6, IL-10, and IL-18 was quantified by real-time PCR. mRNA expression of IFN-α and IFN-β was carried out in the primary cholangiocytes following RRV infection. Total RNA from the tissues and primary cholangiocytes was extracted using the RNeasy Mini Kit (Qiagen, Germantown, MD) according to manufacturer’s instructions. cDNA pools generated using standard reagents (Invitrogen) were subjected to real-time kinetic PCR on a Mx-3000 Multiplex Quantitative PCR (Stratagene, La Jolla, CA) using SYBR Green to quantify mRNA expression of these cytokines relative to glyceraldehyde-3-phosphate dehydrogenase (GAPDH) using techniques previously described [14]. Primers for the assayed cytokines are detailed in Table 1.

**Table 1:** Primers used for real-timer PCR experiments.

Primers	Sequences
IFN-γ	Forward: 5’- GGCTGTCCCTGAAAGAAAGC -3’ Reverse: 5’- GAGCGAGTTATTTGTCATTCGG -3’
TNF-α	Forward: 5’- AAGGGAGAGTGGTCAGGTTGCC -3’ Reverse: 5’- CCTCAGGGAAGAGTCTGGAAAGG -3’
IFN-α	Forward: 5’- GACTTTGGATTTCCCCTGGAG -3’ Reverse: 5’- AAGCCTTTGATGTGAAGAGGTTC -3’
IFN-β	Forward: 5’- TACGTCTCCTGGATGAACTC-3’ Reverse: 5’- TCTTCAAGTGGAGAGCAGTT -3’
IL-6	Forward: 5'- GTCGGAGGCTTAATTACACA -3' Reverse: 5'- TGCATCATCGTTGTTCATAC -3'
IL-10	Forward: 5'- CGCTGTCATCGATTTCTC -3' Reverse: 5'- ATTCATGGCCTTGTAGACAC -3'
IL-18	Forward: 5'- AAATGGAGACCTGGAATCAGAC -3' Reverse: 5'- TTTGTCAACGAAGAGAACTTGG -3'
GAPDH	Forward: 5’- TACACTGAGGACCAGGTTGT -3’ Reverse: 5’- CAAAGTTGTCATTGAGAGCA -3’

### Quantification of IFN-α protein in primary cholangiocytes after RRV infection

Primary cholangiocytes isolated at DOL2 and 9 were seeded in 24-well plates in DMEM/F12 as previously described and incubated at 37°C until 90% confluence. Plates were washed with EBSS, infected with RRV at an MOI of 100, and incubated at 37°C. Supernatants were collected at 16 and 24 hours for IFN-α quantification using the VeriKine Mouse Interferon Alpha ELISA kit (Pestka Biomedical Laboratories, Piscataway, NJ).

### Quantification of live infectious rotavirus

Tissue homogenates and cell suspensions were analyzed for the presence of infectious rotavirus by a fluorescent focus-forming assays (FFA) as previously described [26].

### Measurement of viral binding using an attachment assay

The ability of the virus to attach to the primary cholangiocytes was assessed by binding assays as described previously [27]. In brief, cultured cells were grown to confluence in 24-well plates. Attachment assays were performed in triplicate. At the time of assay, the cells, medium, and inoculating virus were cooled to 4°C. Cells were inoculated with RRV at a multiplicity of infection (MOI) of 0.5 and were incubated for 1 h at 4°C. The inoculum was removed, and the cells were washed twice to remove any unbound virus. The wash fluids and the residual inoculum were combined to account for all unbound viruses. The cells underwent 2 freeze-thaw cycles, and any virus found within the final cell fraction reflected bound virus. The amounts of bound and unbound virus were determined by FFA analysis. The amount of bound virus was expressed as a percentage of the total amount of virus used to inoculate the cells.

### Measurement of RRV replication in primary cholangiocytes

Cholangiocytes isolated from murine livers at different days of life were seeded in 24-well plates in DMEM/F12 as previously described and incubated at 37°C until 90% confluence. Plates were washed with EBSS and infected with RRV at varying MOI at 37°C for 1 h. At an MOI of 1, the amount of virus equals the number of cells. The cultures were washed and incubated with serum free DMEM/F12 at 37°C for 18 h and viral yields were assessed by FFA.

### Statistical analysis

Results of morbidity and mortality from RRV infection were based on at least 20 pups per infection time point. Findings were expressed as percentages of pups expressing symptoms (at least two findings) and percent survival. Analysis of these non-continuous variables was done using Chi-square and Fisher Exact testing. Each temporal subset utilized for ELISA, FFA, and RT-PCR analysis consisted of at least 10 pups. Results of these continuous variables were expressed as mean +/- Standard Error of the Mean (SEM) and analyzed using Student’s t-test and ANOVA with post-hoc testing as appropriate. Biliary injury, in each category, was represented as a median score followed by its range. Comparisons were made using ANOVA on Ranks with Dunn’s Method for post-hoc testing with p values less than 0.05 deemed significant.

## Results

### Initiation of the murine model of BA is temporally dependent

**Figure 1:**
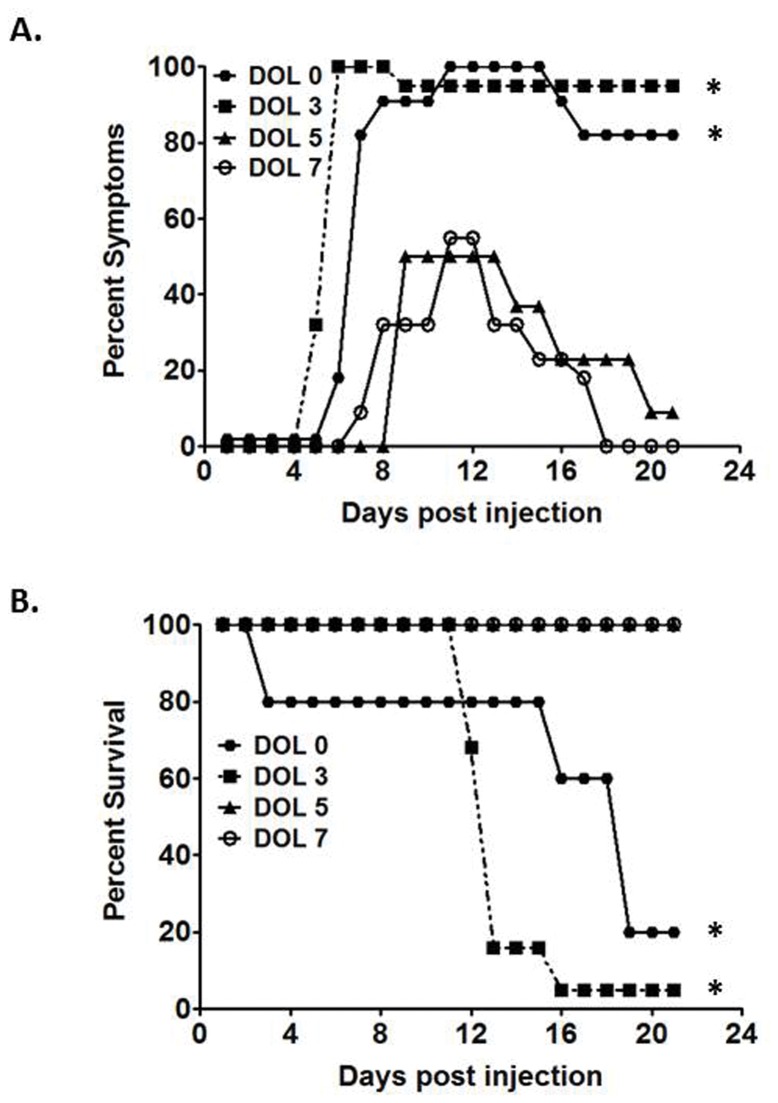
Symptoms and survival after RRV infection. The top graph illustrates morbidity from viral infection on the corresponding day of infection. Significant resolution of symptoms in the late infection groups occurs by day of life (DOL) 21. The bottom graph illustrates mortality. * p<0.05 when compared with both infections on DOL 5 and DOL 7 to infections on DOL 0 and DOL 3.

100% of BALB/c pups challenged with RRV on DOL 0 developed symptoms, and only 20% of mice were alive at 21 days of age. Infection on DOL 3 caused symptoms in 95% of pups with a survival rate of 5%. Because development of symptoms and mortality rates were similar in pups injected on DOL 0 and 3, these two cohorts were categorized as the early infection group. Pups challenged on DOL 5 resulted in symptoms in 50% of pups, and by post injection (PI) day 21, only 9% had manifestations of hepatobiliary disease. All of the inoculated pups survived. In a similar fashion, 55% of pups infected on DOL 7 developed symptoms and, by PI day 21, all were symptom-free and survived (Figure 1). Because pups challenged on DOL 5 and 7 responded similarly, these cohorts were categorizes as the late infection group. When the early and late infection groups were compared, the symptoms of biliary obstruction and mortality in the early infection group were greater than those of the late infection group (p<0.05). Normal saline challenged pups developed no signs of hepatobiliary disease and experienced no mortality. These results confirm the temporal dependence of the murine model of biliary atresia [21].

### Histological biliary injury and elevated liver enzymes correlate with *in vivo* findings

**Figure 2:**
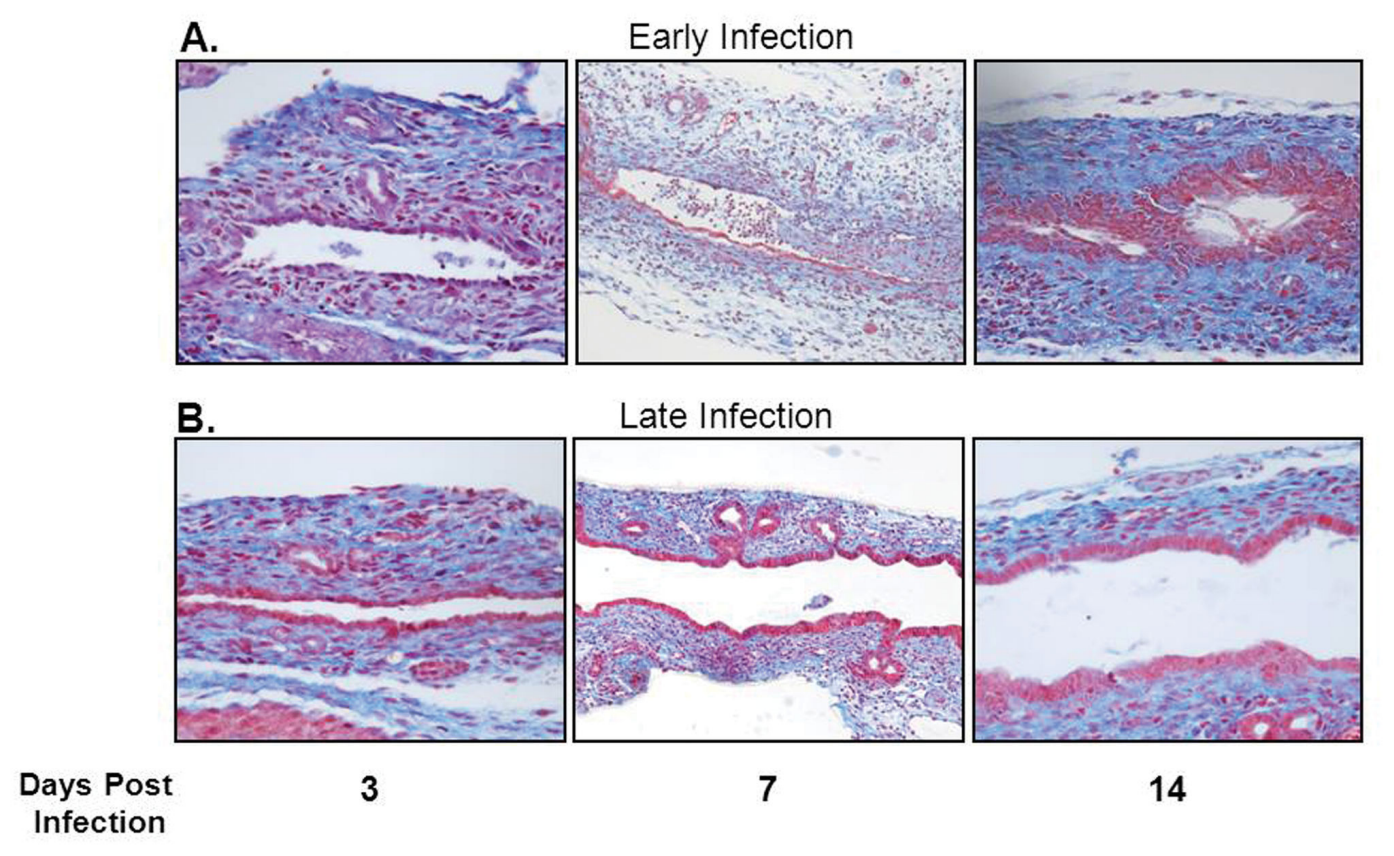
Histologic appearance of the extrahepatic biliary tract following infection with RRV in early versus late injected mice. Mason’s trichrome staining of extrahepatic bile ducts after infection with RRV leads to complete obstruction of extrahepatic biliary duct in early infection group (A). In contrast, the late infection group had some mild inflammation with a patent bile duct (B). In each group, the images are magnified 40X.

**Figure 3:**
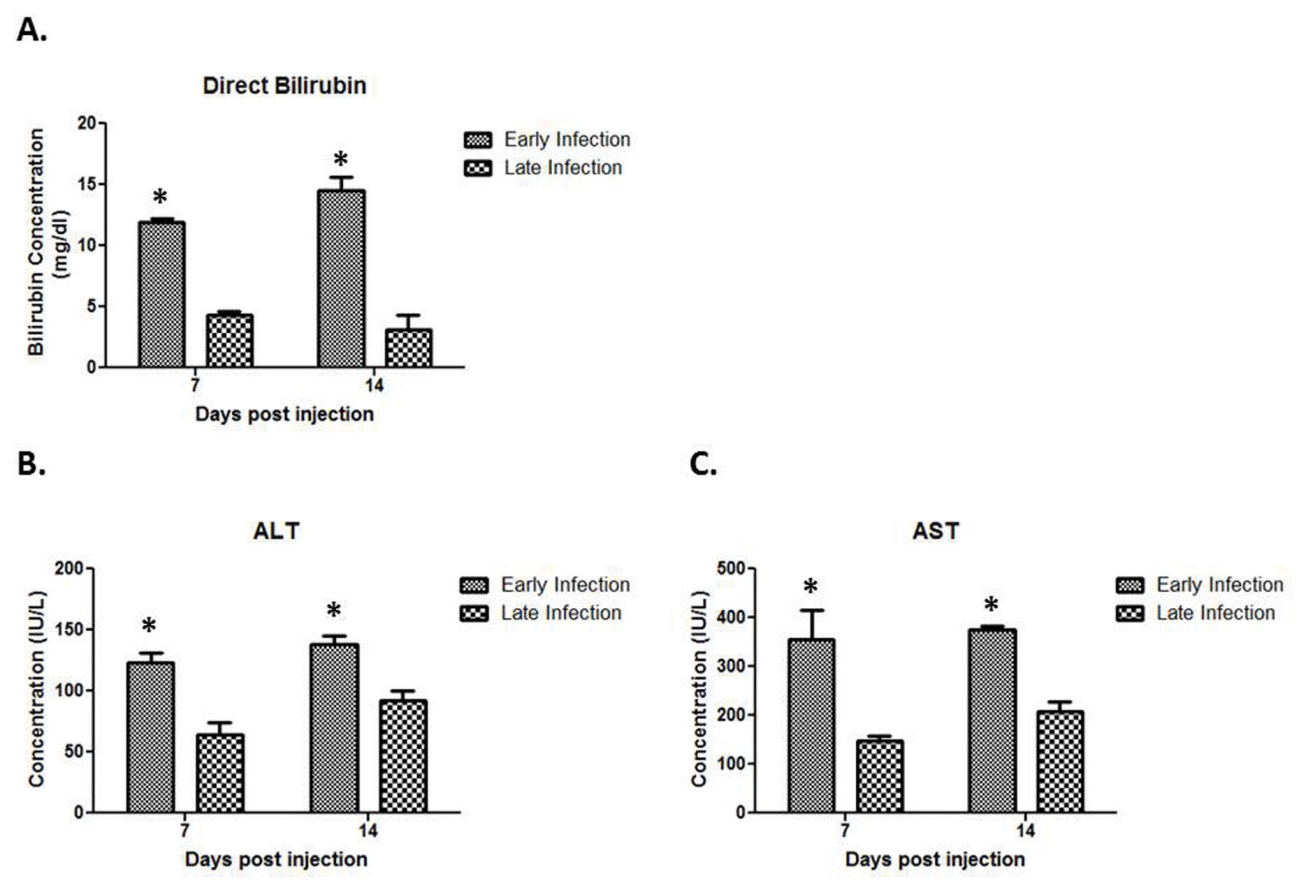
Quantification of Liver Enzymes. Serum harvested from early and late infected pups demonstrates significantly higher concentrations of direct bilirubin, ALT, and AST at both 7 and 14 days post injection *p<0.05.

Histological evaluation of extrahepatic biliary trees demonstrated that biliary epithelial injury was similar between the early and late infection groups over the first five days after RRV challenge; however, when comparing samples harvested 7 days PI, the amount of cholangiocyte epithelial damage and bile duct edema was significantly greater in the early infection group (Table 2). In the early infection group, luminal cholangiocytes were blunted in height, atrophic, and sloughed in various sections of bile ducts. In contrast, the morphology and structure of cholangiocytes in the late infection group were unaffected (Figure 2). By 14 days PI, the early infection group demonstrated greater intraepithelial injury and stromal proliferation, but in late infected mice, the biliary epithelium recovered with resolution of inflammation. Serum harvested from early and late infected pups was examined for direct bilirubin, ALT and AST levels. Significantly elevated levels of direct bilirubin were seen in the early infection group at 7 and 14 days PI as compared to the late infection group (Figure 3A). ALT and AST levels were also significantly increased in the early infection versus the late infection group at 7 and 14 days PI (Figure 3B, C). Levels of direct bilirubinwere significantly elevated over normal saline injected control mice at 7 and 14 days PI in the early infection group and at 7 days PI in the late infection group; ALT and AST levels were significantly higher than the normal saline controls at 14 days PI in the early infection group (data not shown).

**Table 2:** Severity of injury as indicated by histologic assessment to the extrahepatic biliary duct following injection with RRV^a^.

**Days Post Infection**	**Early Infection**	**Late Infection**
**Day 3**		
Intraepithelial Inflammation	1 (0-1)^b^	0 (0-0)
Epithelial Damage	1 (0-2)	0 (0-0)
Edema	1 (0-2)	0.5 (0-1)
Submucosal Inflammation	1.5 (1-3)	1(1-1)
Stromal Proliferation	0 (0-0)	0 (0-0)
**Total Injury**	**5 (4-8)**	**1.5 (1-2)**
**Day 5**		
Intraepithelial Inflammation	1 (0-1)	2 (1-2)
Epithelial Damage	1.5 (1-3)	2.5 (2-3)
Edema	1.5 (1-2)	1.5 (1-2)
Submucosal Inflammation	3 (1-3)	2 (2-3)
Stromal Proliferation	0 (0-0)	0 (0-0)
**Total Injury**	**7 (7-9)**	**9 (8-11)**
**Day 7**		
Intraepithelial Inflammation	2 (1-4)	1 (1-2)
Epithelial Damage	2.5 (2-4) *^c^	1.5 (1-3)
Edema	2 (1-3)*	0 (0-0)
Submucosal Inflammation	2 (1-3)	2 (1-2)
Stromal Proliferation	1 (1-2)	0.5 (0-1)
**Total Injury**	10 (6-16)*	**5 (3-7)**
**Day 12**		
Intraepithelial Inflammation	2 (1-3)*	0 (0-1)
Epithelial Damage	2 (1-3)	1 (1–1)
Edema	0 (0-3)	0 (0-0)
Submucosal Inflammation	2 (1-2)	0 (0-0)
Stromal Proliferation	1 (1-2)*	0 (0-0)
**Total Injury**	7 (4-13)*	**2 (2-3)**

^a^ Severity of bile duct injury after inoculation with the RRV, as determined by histologic appearance of the extrahepatic biliary tract. All sections were graded by a pathologist. ^b^ Injury scoring is based on an ordinal scale from 0 to 4 Values are expressed as median scores, with ranges indicated in parentheses. The maximum total injury score possible was 20 ^c^ *p < 0.05 when compared with late infection.

### Expression of pro-inflammatory genes is increased in early-infected compared to late-infected animals

**Figure 4:**
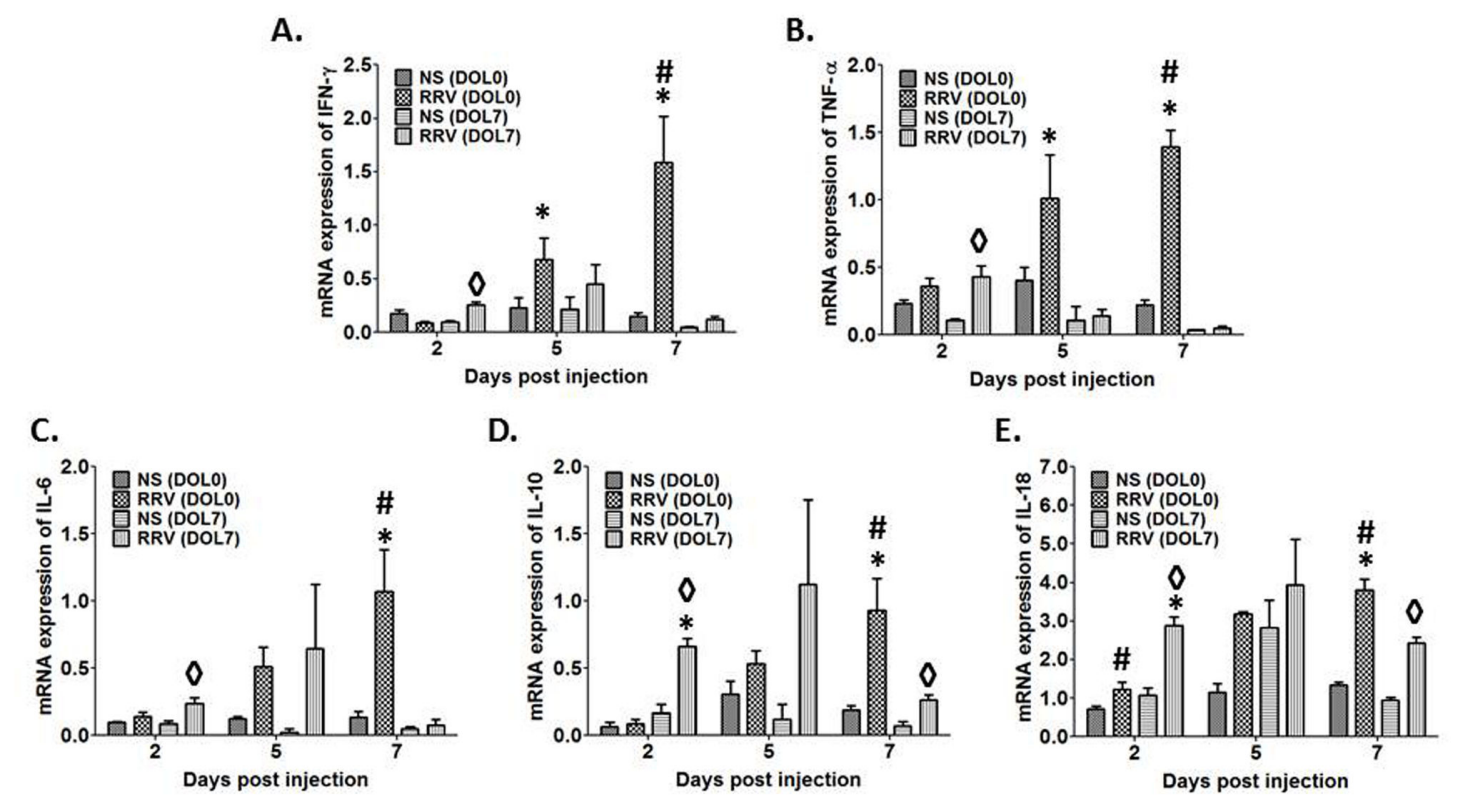
Cytokine mRNA expression after RRV infection on DOL 0 or 7. Expression of IFN-γ (A), TNF-α (B), IL-6 (C), IL-10 (D), and IL-18 (E) mRNA in livers harvested from newborn mice injected with RRV on DOL 0 increases over time as compared to control. In contrast, cytokine levels from livers extracted from mice infected on DOL 7 are more often significantly elevated over control, 2 days post-infection with a general decrease in level as compared to DOL 0 injected mice. Finally, by 7 days post-infection, mRNA expression of all 4 cytokines is higher in mice injected with RRV on DOL 0 as compared to those infected on DOL 7 (* p<0.05 DOL 0 infected vs. DOL 7, # p<0.05 DOL 0 infected vs. uninfected, ◊ p<0.05 DOL 7 infected vs. uninfected).

Given the clinical and histological findings, we determined whether these findings correlated with differences in the production of cytokines previously shown to play a role in the pathogenesis of murine biliary atresia. RT-PCR revealed that mRNA levels of the pro-inflammatory cytokines IFN-γ, TNF-α, IL-6, IL-10, and IL-18 from liver homogenates of animals inoculated with RRV on DOL 0 (representative of early infection) were all significantly elevated compared to those from control animals 7 days after injection (p<0.05) (Figure 4). In contrast, expression of these mRNA in livers from mice injected with RRV on DOL 7 (representative of late infection) was significantly different. IFN-γ, TNF-α, and IL-18 mRNA expressions were elevated 2 days PI (p<0.05); however by PI day 5, expression levels of these cytokines were not significantly different compared to control. By PI day 7, mRNA level of all 3 cytokines in the late infection group had decreased to values below those observed 2 days PI. Interestingly, the mRNA expression of IL-10 in the RRV injected mice was increased at all-time points in both early and late infected mice when compared to controls. In the case of IL-6, mRNA expression levels were not significantly different in late infected animals at any time point.

### Differences in temporal distribution, not viral tropism, of RRV between the early and late infection groups characterize murine BA

**Figure 5:**
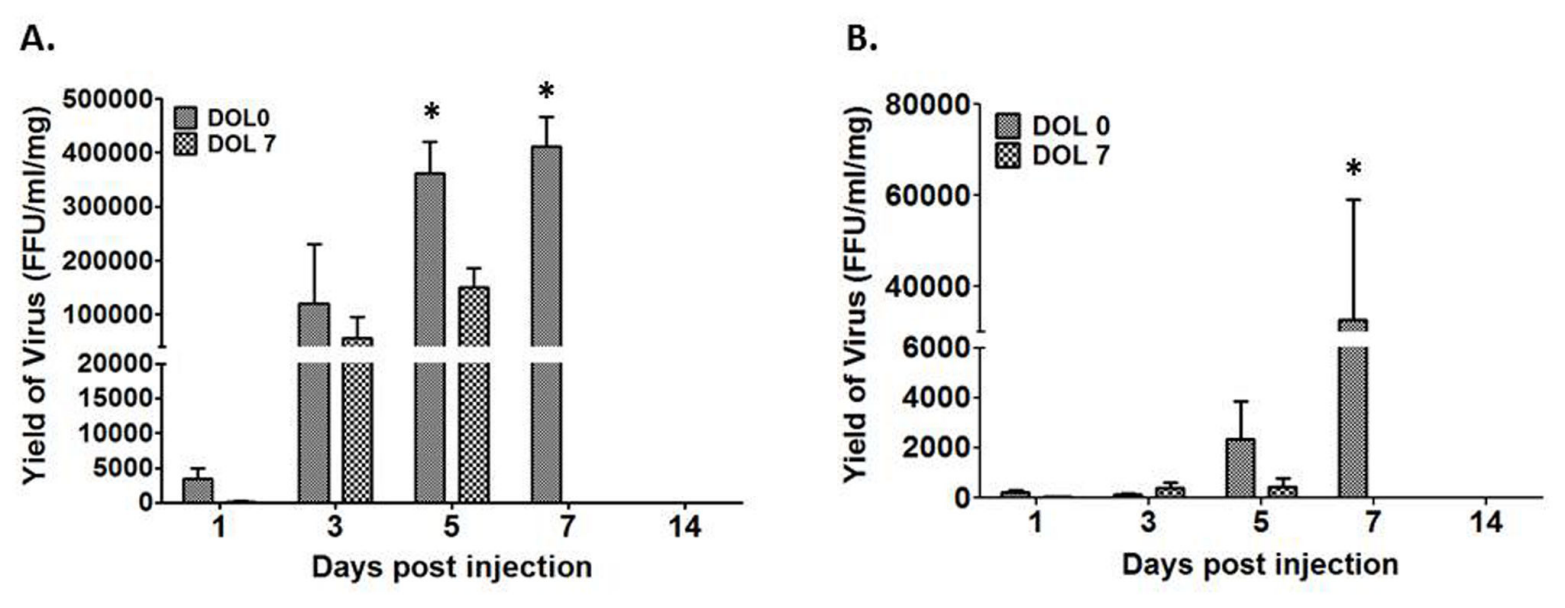
Quantification of infectious RRV post infection. The two graphs illustrate the quantity of live RRV present in the extrahepatic biliary tree (A) and in the liver (B) 7 days post injection. * p<0.05.

To establish the basis for the observed temporal dependence of murine BA, we determined whether the target of viral infection varied according to the DOL on which RRV challenge took place as well as if virus reached the hepatobiliary system in late infection. The presence of live virus was detected in the bile ducts and livers within 24 hours of infection in all RRV-inoculated pups, regardless of early or late inoculation (Figure 5). The peak titer of live virus differed depending upon the DOL on which inoculation took place. At PI day 1 and 3 there was no significant difference in the amount of live virus in either biliary or hepatic samples in both groups. At 7 days PI, there was a greater amount of RRV present in both bile duct and liver samples from the early infection group as compared to those from the late infection group. By day 14 PI, no infectious virus was detectable in either bile duct or liver samples regardless of inoculation period (Figure 5A, B). The amounts of infectious RRV detected in homogenates of the extrahepatic biliary tract were ten-fold higher than liver homogenates from corresponding mice at each time point analyzed (Figure 5A,B).

**Figure 6:**
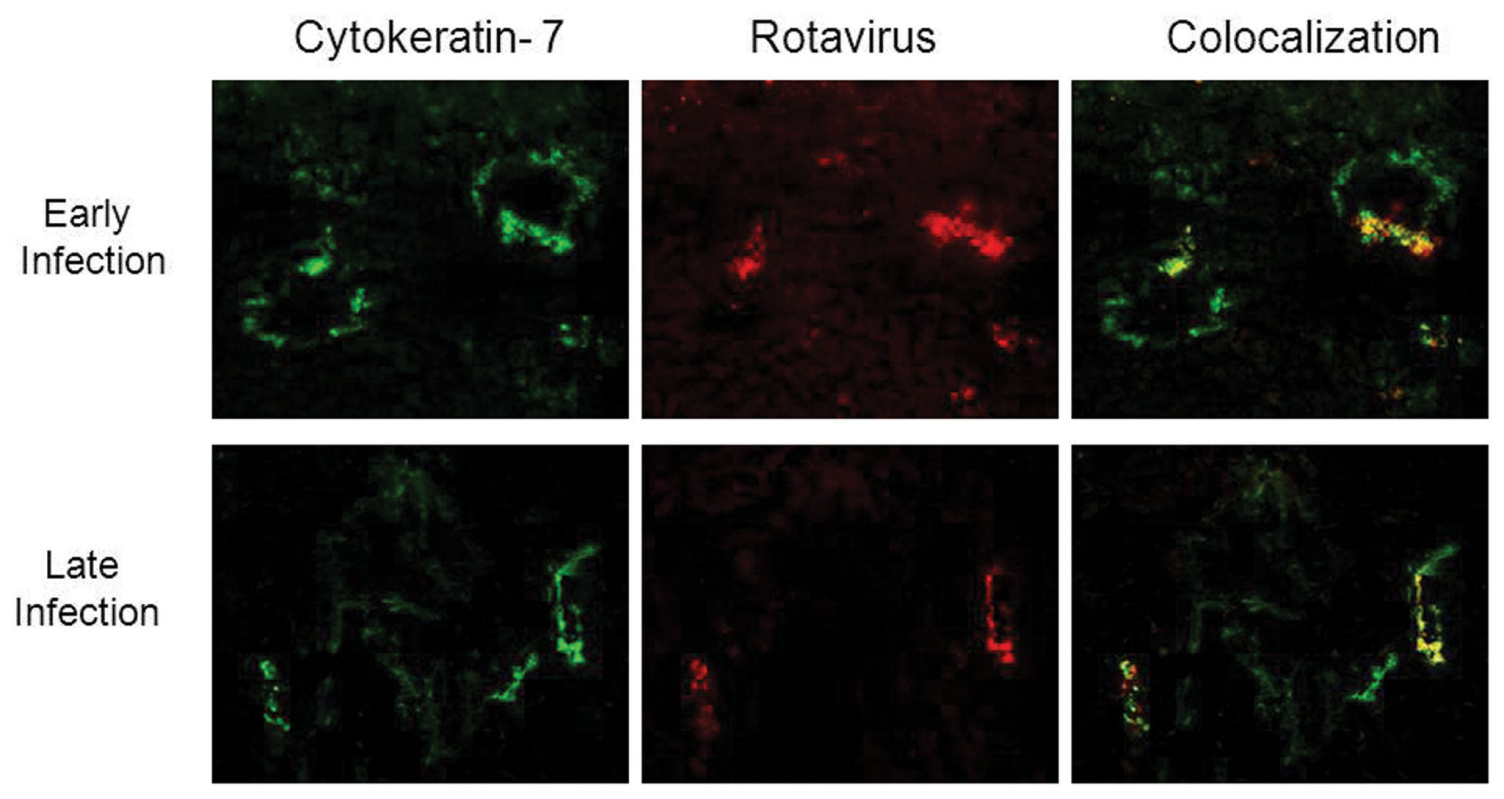
Immunofluorescence of liver 5 days post-RRV infection. Column A demonstrates staining of biliary epithelium (cytokeratin 7 positive, green). Column B demonstrates staining for rotavirus (red). Column C demonstrates overlay images where co localization corresponds to yellow-orange staining. Staining of samples from Day 3 infection is similar to Day 0 and samples from Day 5 infection is similar to Day 7 (data not shown). All images were magnified 20X.

The target of RRV infection was identical regardless of the DOL on which inoculation took place. Immunofluorescence demonstrated co-localization of virus and cytokeratin 7, a marker for biliary epithelium, on PI days 5 and 7 for the early and late infection groups, indicating that the biliary epithelium was the specific target of RRV binding and infection. Representative samples harvested at 5 days PI are shown in Figure 6. Saline controls were negative for RRV staining (data not shown).

### The ability of RRV to bind and infect primary cholangiocytes is determined by developmental age

In order to assess differences of viral yield among cholangiocytes of differing age, we infected primary BALB/c murine cholangiocytes harvested from DOL 2 and 9 mice with RRV at increasing MOIs. As demonstrated by Table 3, the amount of live, infectious RRV produced was significantly higher in cholangiocytes obtained from DOL 2 mice as compared to the amount from cholangiocytes obtained from DOL 9 mice (p<0.05). These differences persisted at all MOI tested. Interestingly, RRV demonstrated no difference in its ability to bind to DOL 2 or 9 cholangiocytes (12.8%±2.2 vs 10.8%± 0.6 respectively) as determined by binding assays.

**Table 3:** In vitro viral yield after infection of primary cholangiocytes with increasing MOI of RRV^a^.

**MOI^b^**	**Average Titer at 18 Hrs Post Infection (x10^5^)^c^**	
	Day 2 ^d^	Day 9
**1**	10.0±0.1^e^ *^f^	3.2±0.1
**30**	41.7±4.7*	25.7±1.2
**300**	1549.3±80.9*	802.7±9.3
**3000**	1558.7±91.9*	961.3±82.6

^a^ 1.3 X 10^6^ Primary cholangiocytes inoculated with RRV to determine replication rate. ^b^ Multiplicity of Infection (MOI) ^c^ Amount of virus was determined by a focus-forming assay. Values are means ±standard errors. ^d^ Day post birth cholangiocytes were harvested from mouse liver. ^e^ Values are expressed as mean FFU/ ml ± standard errors. ^f^ *p<0.05

### Expression of interferon alpha/beta in primary cholangiocytes following RRV infection is temporally dependent

**Figure 7:**
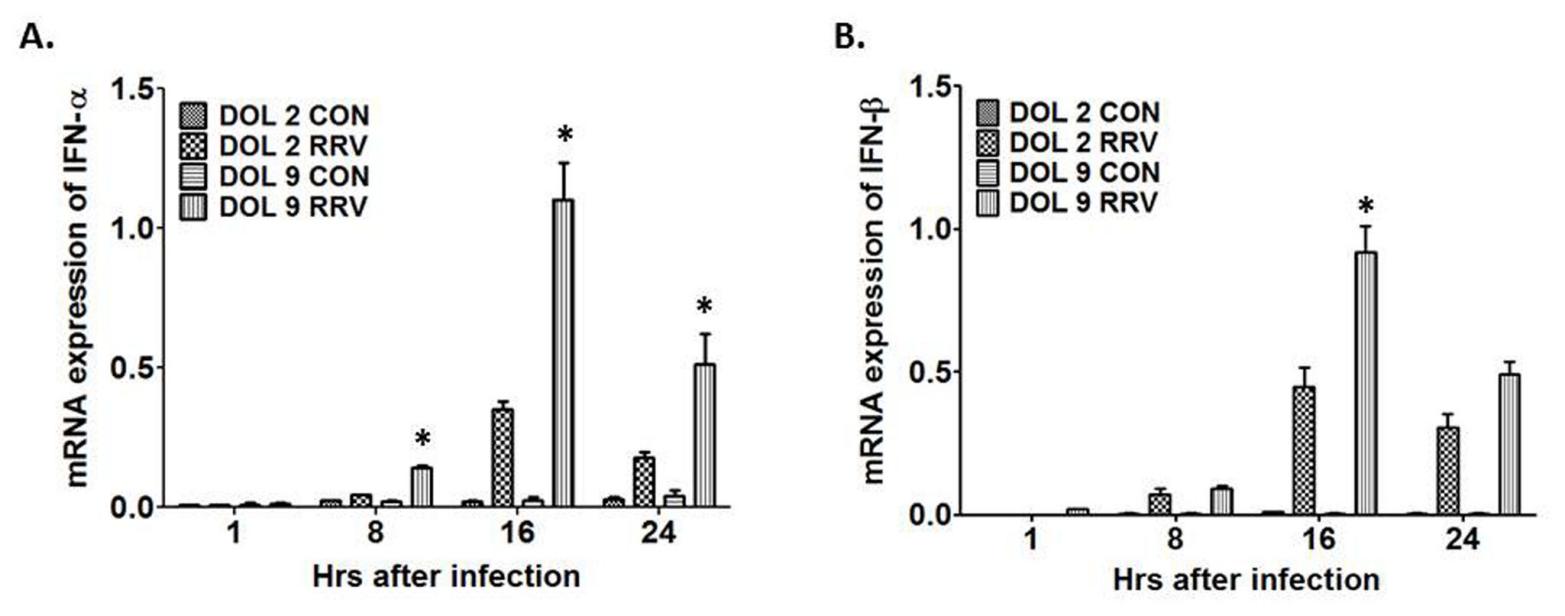
INF-α and INF-β mRNA expression after RRV infection in DOL 2 and DOL 9 primary cholangiocytes. Following RRV infection in the primary cholangiocytes there was a significant increased expression of IFN-α (more than 3 fold) (A) and of IFN-β (2 fold) (B) in the DOL 9 cholangiocytes when compared to DOL 2 cholangiocytes *p<0.05.

The above results indicate that cholangiocytes obtained from DOL 2 and 9 mice allow for similar binding and infectivity of RRV. To determine the basis by which primary cholangiocytes harvested from DOL 2 mice replicate RRV to a higher titer than primary cholangiocytes harvested from DOL 9 mice, we examined the expression of interferon alpha/beta (IFN-α/ β), which plays a key role in the host defense against invading virus. Real-time PCR was used to determine the IFN-α/β gene expression over a 24-hour time course after infection of primary cholangiocytes harvested from DOL 2 versus DOL 9. Following infection the cholangiocytes harvested from DOL 9 mice expressed a 3-fold larger amount of IFN-α compared to cholangiocytes harvested from DOL 2 mice at 8, 16 and 24 hours (Figure 7A). Similarly DOL 9 cholangiocytes express 2-fold higher amount of IFN-β at 16 hours compared to DOL 2 cholangiocytes (Figure 7B).

**Figure 8:**
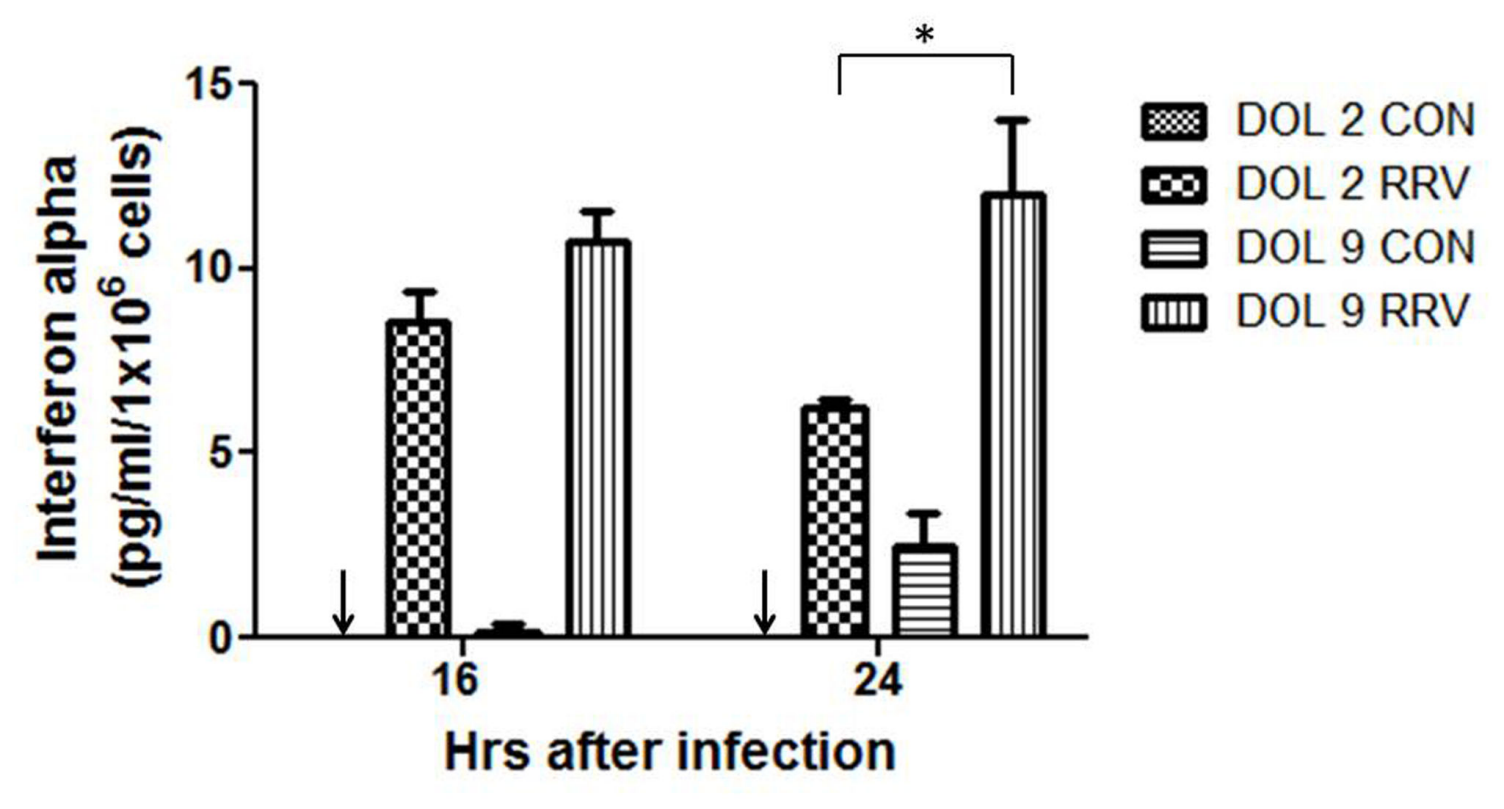
Quantification of IFN-α protein in primary cholangiocytes after RRV infection. Supernatants collected at 16 and 24 hours from DOL 2 and 9 primary cholangiocyte after RRV infection demonstrates greater quantities of IFN-α protein at 16 hr PI with a significantly higher quantity at 24 hours in the DOL 9 cholangiocytes compared to the DOL 2 cholangiocytes *p<0.05. Arrows indicate undetectable levels of interferon alpha.

To confirm the gene expression data interferon alpha protein quantification was also performed on DOL 2 and DOL 9 primary cholangiocytes after RRV infection. Supernatants collected at 16 and 24 hours, which represented the highest level of gene expression on real-time PCR, demonstrated greater quantities of IFN-α protein at both the time points in the DOL 9 cholangiocytes compared to the DOL 2 cholangiocytes (Figure 8). The amount produced at 24 hours in the DOL 9 cholangiocytes was significantly increased over the amount produced by the DOL 2 cholangiocytes, consistent with the findings of gene expression.

### Initiation of the murine model of BA is extended to late infected IFN-α/β receptor deficient mice (IFN-αβR^−/−^)

**Figure 9:**
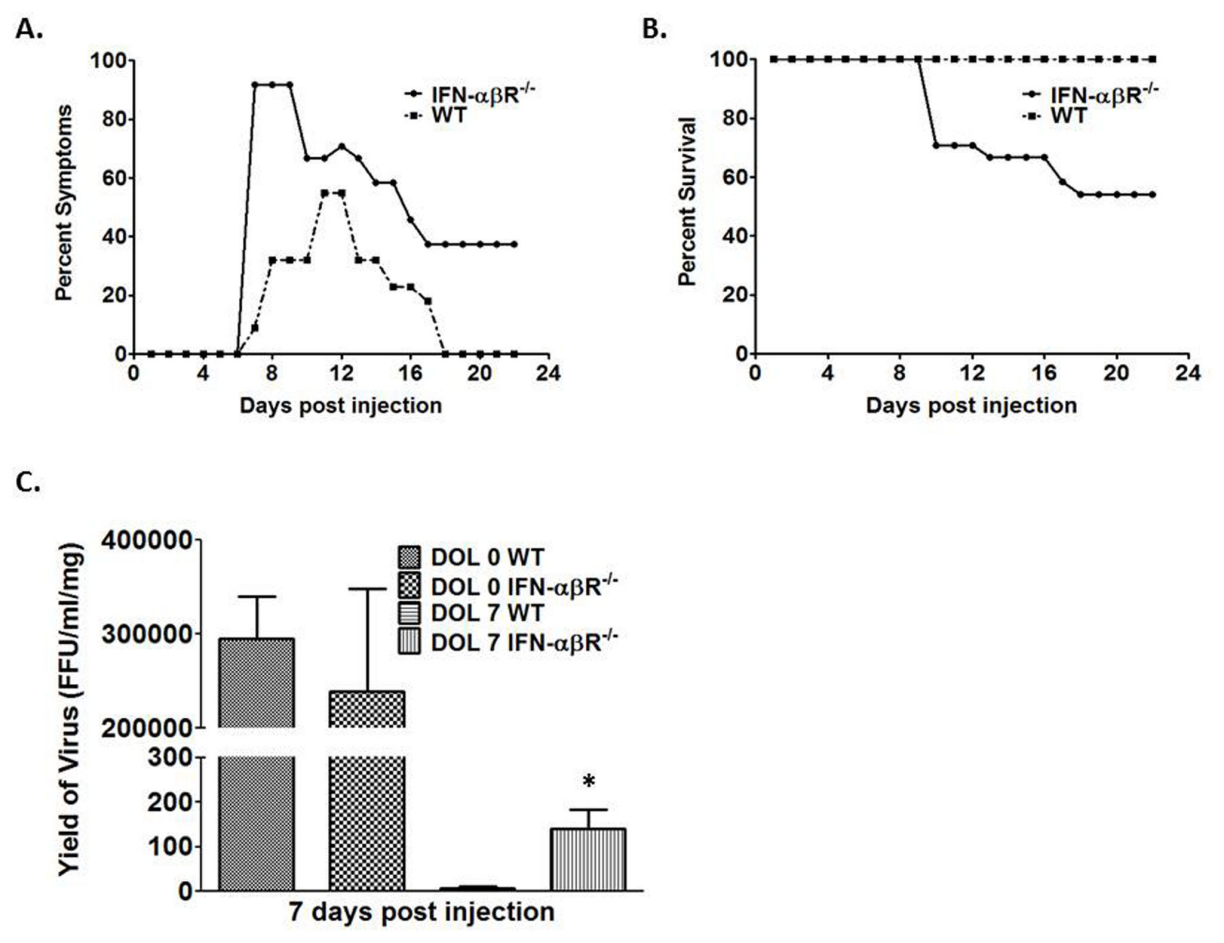
Symptoms, survival and viral titer in IFN-αβR^-/^^-^mice following RRV infection. Graph A illustrates morbidity from viral DOL 7 infected IFN-αβR^−/−^vs WT mice. Graph B illustrates mortality. Graph C demonstrates the quantity of live RRV present in the extrahepatic biliary tree seven days post infection. *p<0.05 when compared between RRV infected IFN-αβR^−/−^mice and WT mice.

To determine if IFN-α regulates the temporal dependency of the murine model of biliary atresia, we injected IFN-αβR^-/-^ mice at DOL 0 and DOL 7. IFN-αβR^-/-^ mice injected at DOL 0 showed symptoms and mortality similar to that of wild-type DOL 0 injected mice (data not shown). In contrast, 79% of IFN-αβR^−/−^mice injected at DOL 7 showed symptoms of biliary disease compared to 55% of wild-type mice (Figure 9A). Twenty-one days post infection DOL 7 injected IFN-αβR^−/−^mice had a 46% mortality rate as compared to 0% for wild-type mice (Figure 9B).

### Quantity of live RRV in bile ducts seven days post injection

Due to the increase in symptom and mortality rates in DOL 7 injected IFN-αβR^−/−^ mice, we wanted to determine if these were related to an increase in viral replication. DOL 0 injected IFN-αβR^−/−^ mice and WT mice express similar titers of RRV in their bile ducts seven days post infection. In contrast, DOL 7 injected IFN-αβR^−/−^mice expressed a significantly greater amount of live RRV in their bile ducts compared to DOL 7 injected WT mice (Figure 9C).

### Bile duct histology of WT and IFN-αβR^-/-^mice following RRV infection

**Figure 10:**
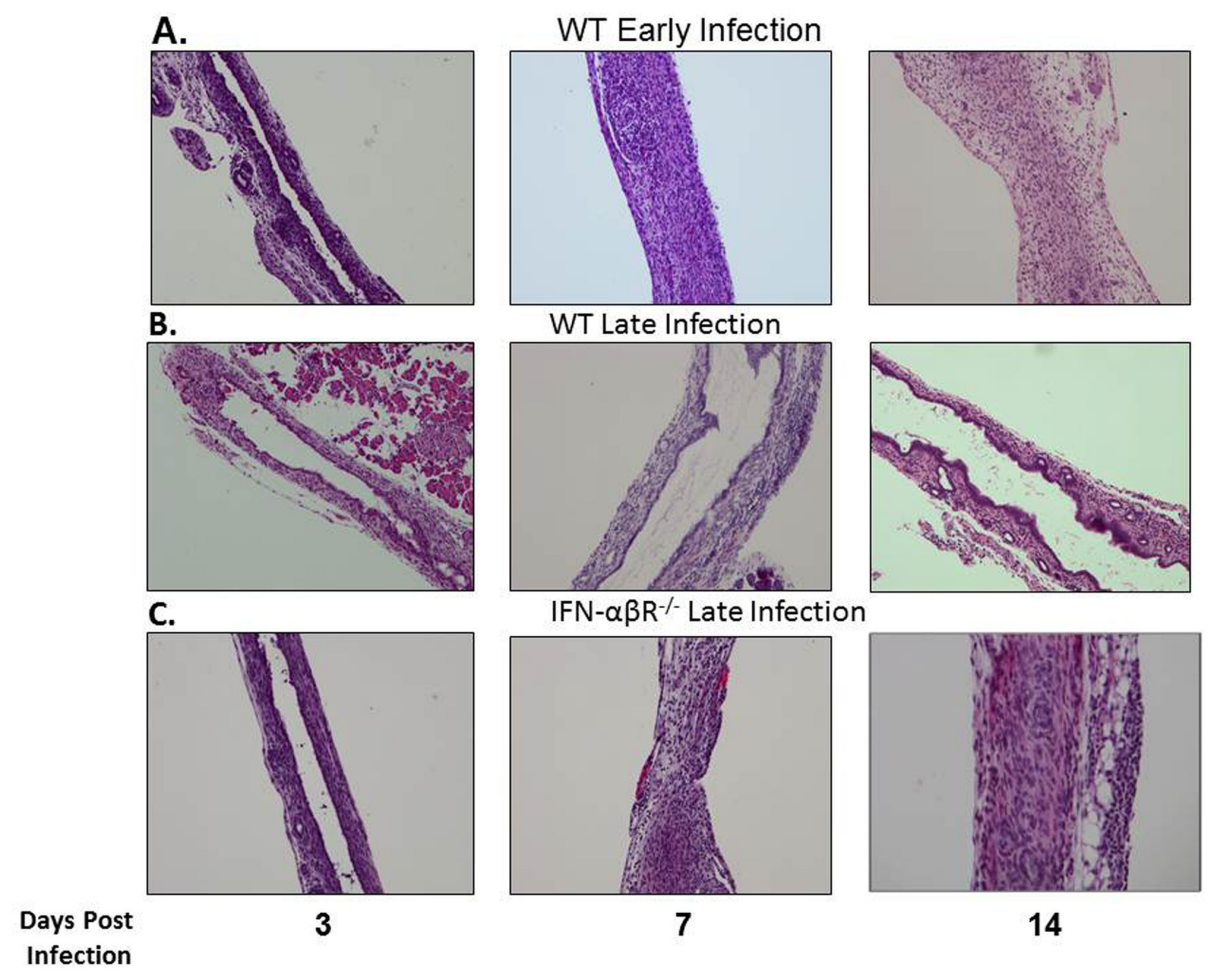
Bile duct histology of WT and IFN-αβR^-/^^-^mice following RRV infection. Extrahepatic bile ducts were harvested from WT and IFN-αβR^−/−^mice 3, 7 and 14 days post inoculation at early and late time points with RRV and stained with H and E. RRV injection led to obstruction of the lumens of the extrahepatic bile ducts in the early but not the late infected WT mice. In contrast, the lumens of the late infected IFN-αβR^−/−^mice were obstructed following RRV injection. Magnification: 10X.

To confirm bile duct obstruction after RRV inoculation of IFN-αβR^−/−^ mice, bile duct histology was examined and compared to WT mice. Obstruction was demonstrated in WT mice in the early but not the late infection group at 7 and 14 days PI. However, IFN-αβR^−/−^ mice in the late infection group demonstrate bile duct obstruction at 7 and 14 days PI, similar to that seen for the WT mice in the early infection group (Figure 10).

## Discussion

Biliary atresia is a disease unique to infancy. Over the first few weeks of life, afflicted children develop progressive biliary obstruction and jaundice. Interestingly, BA does not occur in adolescence or adulthood nor does it recur after liver transplantation. The etiology and underlying factors specific to infancy that govern susceptibility to disease is unknown. One hypothesis is perinatal viral exposure activating both inflammation and an autoimmune response. This is further supported by several reports of de novo autoimmune hepatitis after liver transplantation for BA [28,29]. In parallel to the postulated process occurring in infants, previous studies have demonstrated that induction of hepatobiliary injury in the murine model of BA is dependent on the time of RRV infection [2,21,22]. In early work, Riepenhoff-Talty and Czech-Schmidt delineated the relationship between viral dosage and timing of RRV infection and clinical symptoms [12,20]. To further characterize the temporal dependence of the murine model, we evaluated for differences in the appearance of symptoms and mortality, the ability of RRV to target and replicate in the biliary tract, modifications to the cholangiocyte during cellular development, and activation of the inflammatory cascade, including production of cytokines and chemokines. In this series of experiment, we demonstrate that bile duct obstruction occurs in early but not in the late infected group. These findings are supported by histology as well as an obstructive pattern of liver function tests, including significantly elevated direct bilirubin, ALT and AST levels in the early infection group.

Previously, we established that RRV specifically targets the biliary epithelium for infection in the murine model of BA through cell-surface expression of the α_2_β_1_-integrin [13,15]. In this study, we observe that RRV reaches the biliary tract within 24 hours PI with similar titers of virus present in the biliary samples in the early and late infected groups. Immunofluorescence staining confirmed that the extrahepatic bile duct, specifically the cholangiocyte, was the target of viral infection in all groups. These results suggest that the basis for the temporal dependence of the model is neither viral transport to the biliary tree nor change in the ability of RRV to infect the cholangiocyte. The ability of RRV to replicate within the cholangiocyte, however, was different between the early and late infection groups, with a decrease in the quantity of virus detected in the late group with the progression of time. The amount of virus present may be a factor that governs the temporal dependence of murine BA.

A significant pro-inflammatory state in immature mice driven by cytokines may play an important role in the temporal nature of BA. A number of studies have documented increasing mRNA and protein levels of these cytokines in both humans and mice in relation to disease progression. Up-regulation of pro-inflammatory cytokines in our study corresponds to the temporal pattern of injury as well as the increased yield of live rotavirus. Conversely, it would seem that the level of infectious RRV drives cytokine production based upon results from animals infected later. Early infected mice express higher levels of IFN-γ and TNF-α compared to late infected mice but in contrast, those late infected mice express higher levels of IL-10 at 2 and 5 days PI (Figure 3). Previously, it has been shown that IFN-γ and TNF-α play important roles in the pathogenesis of biliary atresia [14,30]. Since IL-10 is a pro-inflammatory cytokine produced at the site of inflammation, increased IL-10 expression soon after infection in late infected mice might result in less IFN-γ and TNF-α production thereby reducing cholangiocyte damage.

Previous research has suggested that the cholangiocyte may be a critical component in initiating and propagating the pathologic immune response in murine BA [8,31]. The current study suggests that an alteration in cholangiocyte physiology occurs between DOL 1 and 5, thereby modulating cellular response to infection. The observed differences in viral yield from DOL 2 and 9 primary murine cholangiocytes 18 hours after infection would indicate that cellular changes on a genetic or molecular level occur, which influence viral replication. The morphologic and functional heterogeneity of cholangiocytes is comprehensively documented and well-described [32]. On the other hand, the physiologic changes that occur in cholangiocytes as they mature during the neonatal period are not well known and may be logical areas of future study in both murine and human cholangiocytes. From this study we propose that the newborn cholangiocytes infected with virus might have a defective interferon regulatory factor function resulting in lower levels of IFN-α production allowing increased viral replication. It has been reported that cord blood plasmacytoid dendritic cells have an impaired interferon regulatory factor-7 (IRF-7) response leading to defective expression of IFN-α [33]. The same might hold true for early cholangiocytes. To date, there is no data available from epithelial cells.

IFNs are involved in numerous immune interactions acting as inducers, regulators, and effectors of both innate and adaptive immunity. IFN-α**/**β is produced rapidly when viral factors such as envelope glycoproteins or dsRNA interact with cellular pattern-recognition receptors (PRRs), such as toll-like receptors (TLRs), and cytosolic receptors. These host–virus interactions signal downstream to activate transcription factors needed to achieve expression of IFN-α**/**β genes. Among the transcription factors, IRF-7 activation along with IRF-3 and IRF-5 are critical for the induction of type 1 IFNs. IFNs signal mainly through Jak-Stat pathways resulting in induction and activation of numerous intrinsic antiviral factors, such as RNA-activated protein kinase (PKR), the 2-5A system, Mx proteins, and several apoptotic pathways, which provide protection in the neighboring cells against subsequent viral infection.

In this study, we examined the IFN-α/β gene expression over a 24 hour period after infection of primary cholangiocytes harvested from DOL 2 versus DOL 9 mice. The cholangiocytes harvested from day 9 old mice expressed a 3-fold higher amount of IFN-α at 8, 16, and 24 hours and 2-fold higher amount of IFN-β 16 hours post infection compared to cholangiocytes harvested from day 2 old mice. These findings were confirmed by quantification of IFN-α protein at 16 and 24 hours after RRV infection, which demonstrated a significant increase in production by the DOL 9 cholangiocytes over the DOL 2 cholangiocytes. The lack of IFN-α/β production in early cholangiocytes may allow for increased replication rates of RRV. Therefore, we asked the question: if higher IFN-α/β production in the late cholangiocytes reduces RRV replication in vitro and in vivo, will removal of IFN-α/β in the late injected mice make them susceptible to disease? Since IFN-α/β null mice are not available on a BALB/c background, we used the interferon alpha/beta receptor knockout (IFN-αβR^−/−^). When these mice were injected late (DOL 7), symptoms were produced in 79% (50% in WT) with 46% mortality (0% in WT). Histology demonstrated massive liver inflammation with bile duct obstruction similar to DOL 0 injected WT mice.

Recently, Miethke et al. demonstrated the temporal role of regulatory T cells (Tregs) and their control over activated CD8+ and NK cells in murine biliary atresia [23,34]. They found that activated Tregs modulate biliary obstruction while depletion exacerbates disease progression. Although the development of the adaptive immune response contributes to BA pathogenesis, our data suggests that cholangiocyte development also plays an important role in the temporal dependence of murine biliary atresia. The neonatal milieu of decreased interferon as well as the absence of Tregs may provide the necessary environment for sufficient viral load as well as inability to maintain peripheral tolerance and prevent autoimmune disease. Therefore, a search for viral infection in infants with BA may best be sought during the neonatal period, when they have the highest predicted viral burden. While Miethke *et al.* has shown that isolated depletion of Tregs leads to biliary injury, our study suggests that decreased IFN-α/β leads to increased viral load and subsequent induction of the model even in older mice. The development of obstructive cholangiopathy may require these two systems working in parallel, with defects in both the innate and adaptive immune systems early in life.

Further investigation is required to elucidate how the temporal nature of the cholangiocyte response to RRV infection triggers the adaptive immune response. The temporal similarities between murine and human biliary atresia also requires further research. The results of this study documenting early events in the experimental model of BA may supplement clinical understanding of the pathogenesis of human disease and provide targets for early non-surgical intervention.
